# Supplementary material for: Content-rich biological network constructed by mining PubMed abstracts
Source: BMC Bioinformatics. 2004 Oct 8;5:147. doi: 10.1186/1471-2105-5-147 (PMC528731; doi:10.1186/1471-2105-5-147)
Supplement: Additional File 2 — The original results of the above study (non-essential files are deleted to keep the file size under the limit set by BMC bioinformatics). [file 1471-2105-5-147-S2.bz2 › chilibotAdditionalFile2/dip10/630ID8805537E11017/html/PDHD_PDHC.html]

 


 **PDHD** and **PDHC** 
  
Found 6 abstracts in PubMed, retrieved 6.  
 

 What does Google say? 
 PDF only 
| .edu only 

---

**Interactive relationship** (e.g. stimulation, inhibition, etc)

**Neutral relationship**- The E3 gene,  **pdhD** , begins just 4 bp downstream from the stop codon of the  **pdhC**  gene.  Ref: 2253629 Eur J Biochem, 1990
- This is in contrast to the Eukarya and most aerobic bacteria, which use the pyruvate dehydrogenase multienzyme complex  **PDHC** , consisting of multiple copies of three component enzymes E1 pyruvate decarboxylase, E2 lipoate acetyl transferase and E3 dihydrolipoamide dehydrogenase  [ **PDHD** ] , DHLipDH.  Ref: 10832633 Microbiology, 2000

**Non-interactive relationship** (e.g. studied together, co-existance, homology, etc.)

- Whereas Western blots showed that the E3 subunit of  **PDHc**  dihydrolipoamide dehydrogenase  [ **PDHD** ]  did not vary to a large extent under the conditions tested, the E2 subunit dihydrolipoamide acetyltransferase amount followed the trend that was found for the in vitro  **PDHc**  activity.  Ref: 10197995 J Bacteriol, 1999
- The amino acid sequences deduced from the  **pdhC**  and  **pdhD**  genes correspond to proteins of 427 amino acids E2, Mr 4 65 and 469 amino acids E3, Mr 4 93, respectively.  Ref: 2253629 Eur J Biochem, 1990
- Disruptions of the pdhB,  **pdhC** , and  **pdhD**  genes were isolated, but attempts to construct a pdhA mutant were unsuccessful, suggesting that E1alpha is essential.  Ref: 11976308 J Bacteriol, 2002
